# Structural damage progression in patients with early rheumatoid arthritis treated with methotrexate, baricitinib, or baricitinib plus methotrexate based on clinical response in the phase 3 RA-BEGIN study

**DOI:** 10.1007/s10067-018-4221-0

**Published:** 2018-08-04

**Authors:** Désirée van der Heijde, Patrick Durez, Georg Schett, Esperanza Naredo, Mikkel Østergaard, Gabriella Meszaros, Francesco De Leonardis, Inmaculada de la Torre, Pedro López-Romero, Douglas Schlichting, Eric Nantz, Roy Fleischmann

**Affiliations:** 1 Leiden University Medical Center, Albinusdreef 2, 2333 ZA Leiden, The Netherlands; 2 Pôle de Pathologies Rhumatismales Inflammatoires et Systémiques, Institut de Recherche Expérimentale et Clinique, Université Catholique de Louvain and Service de Rhumatologie, Cliniques Universitaires Saint-Luc, Brussels, Belgium; 3 Friedrich-Alexander University Erlangen-Nuremberg and Universitätsklinikum Erlangen, Erlangen, Germany; 4 Hospital Universitario Fundación Jimenez Diaz, Madrid, Spain; 5 Copenhagen Center for Arthritis Research, Center for Rheumatology and Spine Diseases, Rigshospitalet, Glostrup and Department of Clinical Medicine, University of Copenhagen, Copenhagen, Denmark; 6 Eli Lilly & Company, Indianapolis, IN USA; 7 Metroplex Clinical Research Center, University of Texas Southwestern Medical Center, Dallas, TX USA

**Keywords:** Baricitinib, Joint damage, Methotrexate, RA-BEGIN study, Rheumatoid arthritis, Structural progression

## Abstract

The objective of this study was to evaluate structural damage progression based on clinical response in rheumatoid arthritis patients with no or limited prior disease-modifying anti-rheumatic drug treatment receiving the Janus kinase (JAK)1/JAK2 inhibitor baricitinib 4 mg, methotrexate (MTX), or the combination. Data from the phase 3 RA-BEGIN study were analysed *post hoc*. Proportions of patients with structural damage progression (change from baseline greater than the smallest detectable change in modified total Sharp score) at week 52 were evaluated based on sustained Disease Activity Score for 28-joint count with serum high-sensitivity C-reactive protein (DAS28-hsCRP) ≤ 3.2 or Simplified Disease Activity Index (SDAI) score ≤ 11; no formal statistical comparisons between treatments were performed to test these proportions. Baseline factors associated with risk of structural damage progression, including Clinical Disease Activity Index (CDAI) score, were identified using multivariate analysis. Patients achieving versus not achieving sustained DAS28-hsCRP ≤ 3.2 or SDAI score ≤ 11 were less likely to experience structural damage progression at week 52. In patients achieving these responses, structural damage progression was less likely with baricitinib monotherapy or plus MTX than with MTX monotherapy. In patients not achieving these sustained clinical thresholds, structural damage progression was less likely with baricitinib plus MTX than with either monotherapy. Independent of treatment, baseline factors significantly associated with increased risk of structural damage progression included higher hsCRP and CDAI score, smoking, female sex, and lower body mass index. In conclusion, patients achieving versus not achieving sustained DAS28-hsCRP ≤ 3.2 or SDAI score ≤ 11 were less likely to show structural damage progression, irrespective of treatment.

**Electronic supplementary material:**

The online version of this article (10.1007/s10067-018-4221-0) contains supplementary material, which is available to authorized users.

## Introduction

Rheumatoid arthritis (RA) is associated with progressive and irreversible joint damage that starts early in the course of disease and can lead to disability [1]. It is one of the most important reasons to diagnose and effectively treat RA as early as possible, to prevent damage and subsequent functional limitation. In RA, the joint synovium becomes infiltrated with immune cells as a result of a dysregulated immune response [2]. These cells overproduce pro-inflammatory cytokines, such as interleukins, tumour necrosis factor, interferons, various growth factors, granulocyte and macrophage colony-stimulating factors, and chemokines (chemotactic cytokines) [3], which attract further inflammatory and immune cells and stimulate them to release products that cause joint destruction [4]. Consequently, research into new therapies for RA has focused on the targeted blockade of cytokine intracellular signal transduction pathways, such as those involving Janus kinases (JAKs) [5].

Baricitinib is an oral selective inhibitor of JAK1 and JAK2 with less effect on JAK3 and tyrosine kinase 2 [6]. It has been approved in more than 40 countries, including European countries, Japan, and, recently, the USA (2 mg only), at doses of 4 or 2 mg once daily as monotherapy or in combination with methotrexate (MTX) in adults with moderate to severe RA who do not respond adequately or are intolerant to one or more disease-modifying anti-rheumatic drugs (DMARDs) [7, 8]. The efficacy and safety of baricitinib as a treatment for RA were established in four phase 3, randomised, double-blind, multicentre studies in patients with active disease [9–12]. RA-BEGIN was a phase 3, 52-week, double-blind, three-arm, multicentre study assessing the efficacy and safety of oral baricitinib 4 mg once daily as monotherapy or in combination with MTX versus MTX monotherapy in patients with active RA who had no or limited prior DMARD treatment (NCT01711359 [9]). MTX monotherapy, given at a dose of up to 20 mg/week, was the active comparator. Baricitinib 4 mg once daily as monotherapy or in combination with MTX was associated with significant improvements in the signs and symptoms of RA, physical function, and patient-reported outcomes compared to MTX monotherapy. In addition, compared to MTX, structural damage progression was reduced in both baricitinib groups; the difference was statistically significant only for the baricitinib plus MTX group. Further analyses were performed to clarify the effectiveness of baricitinib monotherapy with respect to structural damage progression.

This paper reports the results from *post hoc* analyses conducted using data from the RA-BEGIN study to estimate the proportion of patients with structural damage progression in each treatment group stratified by treatment response (measured using the Disease Activity Score for 28-joint count [DAS28] based on serum high-sensitivity C-reactive protein [hsCRP], DAS28-hsCRP, and the Simplified Disease Activity Index [SDAI]) and to identify baseline factors, including the Clinical Disease Activity Index (CDAI) score, associated with the risk of structural damage progression at week 52.

## Methods

### Patients

Data from patients in the modified intent-to-treat (mITT) population of RA-BEGIN [5] who had both baseline and post-baseline radiographic data were evaluated. Patients aged ≥ 18 years with active RA who had received no or limited treatment with conventional synthetic DMARDs (up to three weekly doses of MTX allowed) and no treatment with biologic DMARDs were randomised to MTX once weekly (*n* = 210), baricitinib 4 mg once daily (*n* = 159) or a combination of baricitinib 4 mg once daily and MTX once weekly (*n* = 215) for 52 weeks. Treatment with MTX was initiated at a dose of 10 mg/week and, if tolerated, increased to 20 mg/week by week 8; a lower dose of MTX was allowed if necessary because of toxicity or at the investigator’s decision. Additional key inclusion criteria were positive anti-cyclic citrullinated peptide antibody (ACPA) > 10 U/mL or rheumatoid factor (RF) > 14 U/mL; ≥ 6/68 tender joints; ≥ 6/66 swollen joints; and hsCRP ≥ 1.2 × upper limit of normal (≥ 3.6 mg/L; normal < 3 mg/L).

### Radiographs

Structural joint damage was measured with radiographs of the hands and feet performed within 4 weeks prior to baseline and at weeks 12, 24, and 52, or at the last visit in the event of early study termination. Joint damage was measured using the van der Heijde-modified total Sharp score (mTSS) [13, 14]. Radiographs were scored centrally and independently by two readers who were blinded to the chronological order of the radiographs, patient identifiers, and treatment groups, with adjudication by a third reader if there was disagreement beyond a predefined level. The mean score from the two readers was used unless the adjudicator provided a score, in which case the two scores in closest agreement were used.

Data for mTSS at week 52 were imputed using linear extrapolation for patients who discontinued the study before week 52, had missing data, or received rescue therapy at week 24 (or any time thereafter). Linear extrapolation used baseline data and the most recent radiographic data before discontinuation, the missed radiograph, or initiation of rescue therapy. Missing post-baseline values were imputed only if a baseline value and a post-baseline value from week 12 onwards were available, and the patient was receiving the same treatment at each applicable time point.

### Structural damage progression

Structural damage progression was defined as change from baseline greater than the smallest detectable change (SDC) in mTSS at week 52. The SDC is the minimum amount of change in a patient’s score that can be assessed beyond measurement error. It was calculated according to the method of Bruynesteyn et al. [15]. The SDC in mTSS in the RA-BEGIN study at week 52 was 1.4.

### Treatment response

Treatment response was measured using the DAS28-hsCRP and the SDAI. Patients were classified into two groups based on their DAS28-hsCRP response: patients in DAS28-group A had a sustained DAS28-hsCRP of ≤ 3.2 at weeks 16, 20, and 24, whereas patients in DAS28-group B had a DAS28-hsCRP of > 3.2 or missing data at any of weeks 16, 20, and 24. The cut-off value of ≤ 3.2 for DAS28-hsCRP was selected when the study protocol was initially designed and before it was understood that low disease activity (LDA) according to DAS28(CRP) is actually lower than 3.2 [16, 17]. For this reason, we do not refer to the value of DAS28-hsCRP < 3.2 as LDA. Supportive analyses were also performed, in which patients were classified into two groups based on SDAI response: patients in SDAI-group A had a sustained SDAI score of ≤ 11 at weeks 16, 20, and 24, whereas patients in SDAI-group B had an SDAI score of > 11 or missing data at any of weeks 16, 20, and 24. The cut-off value of ≤ 11 for SDAI was that recommended by the ACR [18, 19] and EULAR [20, 21] for defining LDA.

Graphical displays (heatmaps) were produced to show individual patient DAS28-hsCRP and SDAI values (by rows) at each visit (by columns) for the different treatments and response groups. These displays use different colours to indicate different response categories: DAS28-hsCRP ≤ 2.6, > 2.6 to ≤ 3.2, > 3.2 to ≤ 5.1, or > 5.1, and SDAI remission, or low, moderate, or high disease activity.

### Analyses

Baseline patient demographic characteristics were grouped by response to treatment (DAS28-group A vs DAS28-group B; SDAI-group A vs SDAI-group B). To estimate the response to treatment, odds ratios (ORs) with the corresponding 95% confidence intervals (CI) and *p* values for the likelihood of a sustained DAS28-hsCRP of ≤ 3.2 or SDAI score ≤ 11 were calculated using a logistic regression model including treatment group and adjusted for the two stratification factors used at randomisation: region + baseline joint erosion status.

To determine the effect of treatment on structural damage progression, ORs (with corresponding 95% CIs and *p* values) for the likelihood of structural damage progression were calculated using a logistic regression model including treatment group and adjusted for the same two stratification factors used at randomisation.

### Structural damage progression according to treatment response

Observed and adjusted proportions of patients in each treatment arm with change from baseline in mTSS > SDC at week 52 were determined for the defined response groups. No formal statistical comparisons were performed between treatments with respect to the proportion of patients with structural damage progression based on treatment response.

Adjusted (least squares [LS] means) estimates of the proportions of patients with change in mTSS > SDC at week 52 were obtained using a multivariate logistic model including the following factors: treatment, response to treatment, age (years), sex, body mass index (BMI), RA duration from diagnosis (years), smoker (yes/no), baseline ACPA (> 10 U/mL positive), baseline RF (> 14 IU/mL positive), baseline hsCRP, presence of radiographic erosions at baseline (yes/no, 0 vs > 0), baseline mTSS, baseline disease status (DAS28-hsCRP or SDAI in the analyses for DAS28-hsCRP and SDAI, respectively), baseline Health Assessment Questionnaire-Disability Index (HAQ-DI) score, geographic location, and treatment-by-response to treatment interaction. Adjusted (LS) means were estimated from the multivariate logistic regression model with continuous covariates fixed at their mean values and categorical covariates fixed at their proportional distribution in the data. Patients with missing data for the covariates used in the model were excluded from multivariate analyses.

### Baseline factors associated with structural damage progression

Associations between baseline factors and structural damage progression were also assessed using a multivariate logistic regression model including the same covariates as in the model above, excluding response to treatment and treatment-by-response to treatment interaction. In addition, to assess whether baseline disease status was associated with structural progression, the CDAI score was included in the model; this score was used because hsCRP was also included. Patients with missing data for the covariates used in the model were excluded from multivariate analyses.

#### Data availability

The data that support the findings of this study are available from Eli Lilly and Company but restrictions apply to the availability of these data.

## Results

### Structural damage progression

Of the 584 patients in the mITT population of RA-BEGIN, 545 had radiographic data. Data from 39 patients with completely missing radiographic data were excluded from analyses (18 in the MTX group, 5 in the baricitinib monotherapy group, and 16 in the combination therapy group). All of the patients with missing radiographic data would have been classified in DAS28- or SDAI-group B apart from one patient receiving baricitinib monotherapy. According to the study protocol, up to three doses of MTX were allowed prior to randomisation; no other previous conventional synthetic DMARDs were allowed. Few of the 584 patients in the mITT population of RA-BEGIN had prior limited exposure to conventional synthetic DMARDs (20/210 in the MTX group, 13/159 in the baricitinib monotherapy group, and 18/215 in the combination therapy group). Of these patients, only one in the MTX group and one in the combination therapy group had missing radiographic data. Of the 545 patients with radiographic data, 85 (15.6%) had a change from baseline in mTSS > SDC at week 52: 21.9% (42/192) of MTX-treated patients, 14.9% (23/154) of baricitinib monotherapy-treated patients, and 10.1% (20/199) of baricitinib plus MTX-treated patients.

ORs for the likelihood of structural damage progression were 0.62 (95% CI 0.35, 1.09) for baricitinib monotherapy versus MTX and 0.39 (95% CI 0.22, 0.70) for baricitinib plus MTX versus MTX.

### Treatment response based on DAS28-hsCRP

A total of 212 patients were classified in DAS28-group A and 372 in DAS28-group B. Baseline characteristics of these patient groups are shown in Table 1. Heatmap plots showing individual responses to treatment over time in the two DAS28-hsCRP groups are presented in Online Resource 1. ORs for a sustained DAS28-hsCRP ≤ 3.2 were 2.8 (95% CI 1.7, 4.4) for baricitinib monotherapy versus MTX and 3.3 (95% CI 2.2, 5.1) for baricitinib plus MTX versus MTX.

**Table 1:** Patient baseline characteristics grouped by DAS28-hsCRP and treatment

	DAS28-Group A (*N* = 212)			DAS28-Group B (*N* = 372)		
Treatment (*N*; % of treatment group)	MTX (*N* = 45; 21.4%)	Bari (*N* = 67; 42.1%)	Bari + MTX(*N* = 100; 46.5%)	MTX (*N* = 165; 78.6%)	Bari (*N* = 92; 57.9%)	Bari + MTX (*N* = 115; 53.5%)
Age, (years)	52 ± 14	52 ± 13	46 ± 14	50 ± 13	50 ± 13	51 ± 13
Female	31 (68.9)	49 (73.1)	71 (71.0)	117 (70.9)	72 (78.3)	85 (73.9)
BMI, (kg/m^2^)	25.1 ± 5.1	27.5 ± 7.3	25.2 ± 5.1	26.9 ± 6.4	26.4 ± 6.1	27.8 ± 6.4
Smoker	7 (15.6)	16 (23.9)	21 (21.0)	41 (24.8)	21 (22.8)	26 (22.6)
Duration of RA from diagnosis, (years)^a^	0.2 (0.1, 0.3)	0.2 (0.1, 0.5)	0.2 (0.1, 1.0)	0.2 (0.1, 0.7)	0.4 (0.1, 1.4)	0.3 (0.1, 1.0)
ACPA positive^b^	41 (91.1)	63 (94.0)	94 (94.0)	152 (92.7)	79 (85.9)	98 (85.2)
RF positive^c^	43 (95.6)	67 (100.0)	94 (94.0)	160 (97.0)	88 (95.7)	110 (95.7)
≥ 1 joint erosion	28 (62.2)	43 (64.2)	60 (60.0)	110 (67.1)	62 (67.4)	77 (67.0)
hsCRP (mg/L)	14.5 ± 10.8	20.5 ± 19.7	25.2 ± 27.5	24.5 ± 23.5	26.1 ± 30.0	23.5 ± 31.1
DAS28-hsCRP	5.5 ± 0.9	5.7 ± 0.9	5.8 ± 1.0	5.9 ± 1.0	6.1 ± 1.0	6.0 ± 0.9
SDAI	37.7 ± 12.6	38.6 ± 12.0	40.0 ± 13.2	42.7 ± 14.1	45.6 ± 14.6	45.2 ± 13.0
mTSS	10.2 ± 23.5	11.8 ± 28.5	9.7 ± 17.5	12.3 ± 21.8	14.4 ± 26.0	13.1 ± 22.3
HAQ-DI total score	1.5 ± 0.7	1.6 ± 0.7	1.5 ± 0.7	1.7 ± 0.7	1.7 ± 0.7	1.6 ± 0.6

Data are reported as mean ± standard deviation or *n* (%) unless otherwise indicated. DAS28-group A: sustained DAS28-hsCRP ≤ 3.2 at weeks 16, 20, and 24; DAS28-group B: DAS28-hsCRP > 3.2 or missing data at any of weeks 16, 20, and 24

^a^Data presented as medians with interquartile range

^b^ACPA positive (> ULN [ULN = 10 U/mL])

^c^RF positive (> ULN [ULN = 14 U/mL])

*ACPA* anti-cyclic citrullinated peptide antibody, *Bari* baricitinib, *DAS28* Disease Activity Score for 28-joint counts, *BMI* body mass index, *HAQ-DI* Health Assessment Questionnaire-Disability Index, *hsCRP* high-sensitivity C-reactive protein, *mTSS* van der Heijde-modified total Sharp score, *MTX* methotrexate, *RA* rheumatoid arthritis, *RF* rheumatoid factor, *SDAI* Simplified Disease Activity Index, *ULN* upper limit of normal

### Structural damage progression based on achieving DAS28-hsCRP ≤ 3.2

**Fig. 1:**
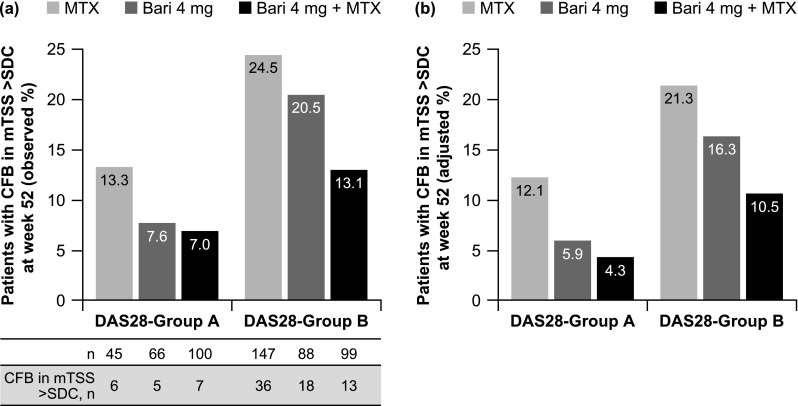
**a** Observed and **b** adjusted proportions of patients with structural damage progression (CFB in mTSS > SDC) at week 52 in DAS28-group A (sustained DAS28-hsCRP ≤ 3.2 at weeks 16, 20, and 24) and DAS28-group B (DAS28-hsCRP > 3.2 or missing data at any of weeks 16, 20, and 24). Adjusted proportions (LS means) were estimated using a multivariate logistic regression model (41 patients were excluded due to missing data for covariates used in the model). *Bari* baricitinib, *CFB* change from baseline, *DAS28-hsCRP* Disease Activity Score for 28-joint counts based on high-sensitivity C-reactive protein, *LS* least squares, *mTSS* van der Heijde-modified total Sharp score, *MTX* methotrexate, *SDC* smallest detectable change (1.4 in the RA-BEGIN-modified intent-to-treat population)

Across treatment groups, smaller proportions of patients who achieved sustained DAS28-hsCRP ≤ 3.2 (DAS28-group A) had structural damage progression at week 52 than patients who did not achieve sustained DAS28-hsCRP ≤ 3.2 (DAS28-group B) (Fig. 1a). The proportion of patients with structural damage progression was lower in the groups with sustained DAS28-hsCRP ≤ 3.2 with baricitinib, either as monotherapy or in combination with MTX, than in the group who achieved sustained DAS28-hsCRP ≤ 3.2 with MTX alone. In patients who did not achieve sustained DAS28-hsCRP ≤ 3.2, structural damage progression was less frequent with combination therapy than with baricitinib monotherapy or MTX monotherapy.

After controlling for potential imbalances with respect to baseline factors that might act as confounders, estimated adjusted (LS) means for the proportions of patients with structural damage progression in DAS28-groups A and B followed a similar pattern to that observed with no adjustment (Fig. 1b). The only comparisons that showed a significantly reduced risk of structural progression were for patients responding to baricitinib monotherapy versus those not responding to baricitinib monotherapy (OR 0.32; 95% CI 0.11, 0.99; *p* = 0.048) and for patients not responding to baricitinib plus MTX versus those not responding to MTX (OR 0.44; 95% CI 0.21, 0.92; *p* = 0.030) (Table 2).

**Table 2:** Odds of structural damage progression based on DAS28-hsCRP and SDAI score with treatment

	DAS28-hsCRP ≤ 3.2		SDAI score ≤ 11	
	Odds ratio (95% CI)	*p* value	Odds ratio (95% CI)	*p* value
In patients who achieved a sustained outcome				
Baricitinib 4 mg vs MTX	0.46 (0.12, 1.71)	0.247	0.42 (0.13, 1.35)	0.145
Baricitinib 4 mg + MTX vs MTX	0.32 (0.09, 1.12)	0.076	**0.25** (**0.08**, **0.80**)	**0.019**
In patients who did not achieve a sustained outcome				
Baricitinib 4 mg vs MTX	0.72 (0.36, 1.45)	0.356	0.70 (0.34, 1.43)	0.327
Baricitinib 4 mg + MTX vs MTX	**0.44** (**0.21**, **0.92**)	**0.030**	**0.42** (**0.20**, **0.89**)	**0.024**
In all patients				
MTX (sustained outcome: yes vs no)	0.51 (0.18, 1.41)	0.192	0.79 (0.33, 1.90)	0.604
Baricitinib 4 mg (sustained outcome: yes vs no)	**0.32** (**0.11**, **0.99**)	**0.048**	0.48 (0.16, 1.39)	0.176
Baricitinib 4 mg + MTX (sustained outcome: yes vs no)	0.38 (0.13, 1.06)	0.065	0.48 (0.17, 1.35)	0.166

Odds ratios were estimated using a multivariate logistic regression model adjusted for baseline factors. Comparisons significantly associated with a decreased risk of structural damage progression are shown in bold

*DAS28-hsCRP* Disease Activity Score for 28-joint counts based on high-sensitivity C-reactive protein, *CI* confidence interval, *MTX* methotrexate, *SDAI* Simplified Disease Activity Index

### Treatment response based on SDAI

When classified by whether or not patients achieved a sustained SDAI score of ≤ 11, 209 patients were included in SDAI-group A and 375 in SDAI-group B. Baseline characteristics of these patients were similar to those of patients grouped according to whether or not they achieved DAS28-hsCRP ≤ 3.2 (Online Resource 2). Heatmap plots showing individual responses to treatment in the two SDAI groups are presented in Online Resource 3. ORs for a sustained SDAI ≤ 11 were 1.9 (95% CI 1.2, 3.0) for baricitinib versus MTX and 2.4 (95% CI 1.6, 3.6) for baricitinib plus MTX versus MTX.

### Structural damage progression based on achieving SDAI ≤ 11

**Fig. 2:**
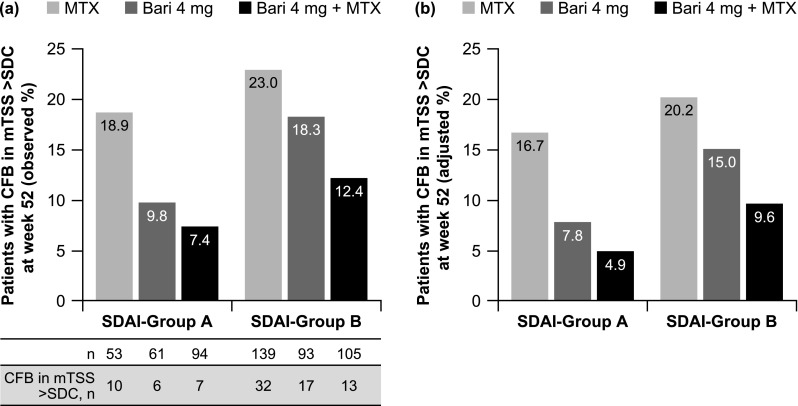
**a** Observed and **b** adjusted proportions of patients with structural damage progression (CFB in mTSS > SDC) at week 52 in SDAI-group A (sustained SDAI score ≤ 11 at weeks 16, 20, and 24) and SDAI-group B (SDAI score > 11 or missing data at any of weeks 16, 20, and 24). Adjusted proportions (LS means) were estimated using a multivariate logistic regression model (47 patients were excluded due to missing data for covariates used in the model). *Bari* baricitinib, *CFB* change from baseline, *LS* least squares, *mTSS* van der Heijde-modified total Sharp score, *MTX* methotrexate, *SDAI* Simplified Disease Activity Index, *SDC* smallest detectable change (1.4 in the RA-BEGIN-modified intent-to-treat population)

Structural damage progression results based on achieving a sustained SDAI score of ≤ 11 were similar to those observed based on achieving DAS28-hsCRP ≤ 3.2 (Fig. 2a). After controlling for potential imbalances with respect to baseline factors that might act as confounders, estimated adjusted (LS) means for the proportions of patients with structural damage progression in SDAI-groups A and B followed a similar pattern to that observed with no adjustment (Fig. 2b). The only comparisons that showed a significantly reduced risk of structural damage progression were for patients responding to baricitinib plus MTX versus those responding to MTX (OR 0.25; 95% CI 0.08, 0.80; *p* = 0.019) and for patients not responding to baricitinib plus MTX versus those not responding to MTX (OR 0.42; 95% CI 0.20, 0.89; *p* = 0.024) (Table 2).

### Baseline factors associated with structural damage progression

**Fig. 3:**
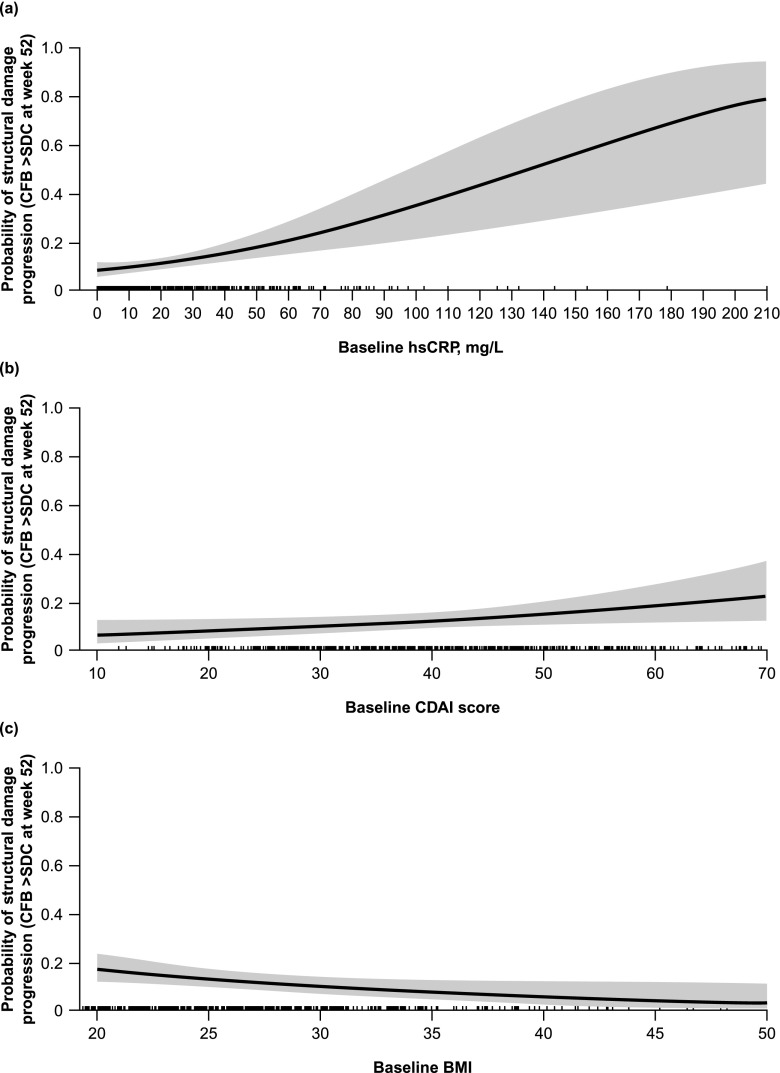
Adjusted probability of structural damage progression as a function of (**a**) baseline hsCRP, (**b**) baseline CDAI score, and (**c**) baseline BMI, estimated using a multivariate logistic regression model. *BMI* body mass index, *CFB* change from baseline, *CDAI* Clinical Disease Activity Index, *hsCRP* high-sensitivity C-reactive protein, *SDC* smallest detectable change (1.4 in the RA-BEGIN-modified intent-to-treat population)

Baseline factors showing a statistically significant association with an increased risk of structural damage progression were higher hsCRP, higher CDAI score, female sex, smoking, and lower BMI (Table 3). The estimated OR for baseline hsCRP was 1.02 (*p* < 0.001), meaning the odds of structural damage progression increased by a factor of 1.02 when baseline hsCRP increased by one unit and other variables remained fixed (Fig. 3a). The estimated OR for baseline CDAI score was 1.03 (*p* = 0.038), meaning the odds of structural damage progression increased by a factor of 1.03 when baseline CDAI score increased by one unit and other variables remained fixed (Fig. 3b). The estimated OR for baseline BMI was 0.94 (*p* = 0.025), meaning the odds of structural damage progression changed by a factor of 0.94 when baseline BMI increased by one unit and other variables remained fixed (Fig. 3c).

**Table 3:** Odds of structural damage progression for different factors

	Odds ratio	Lower 95% CI	Upper 95% CI	*p* value
Baricitinib 4 mg vs MTX	0.57	0.31	1.04	0.066
Baricitinib 4 mg + MTX vs MTX	**0.32**	**0.17**	**0.61**	**< 0.001**
Age	1.00	0.98	1.02	0.820
Sex (female vs male)	**2.28**	**1.17**	**4.44**	**0.015**
Duration of RA	0.95	0.87	1.05	0.322
Baseline BMI	**0.94**	**0.89**	**0.99**	**0.025**
ACPA positive (vs negative)	1.19	0.42	3.41	0.745
RF positive (vs negative)	1.76	0.20	15.06	0.608
Smoker (yes vs no)	**1.92**	**1.04**	**3.56**	**0.037**
Baseline hsCRP	**1.02**	**1.01**	**1.03**	**< 0.001**
Baseline HAQ-DI	0.70	0.45	1.09	0.111
Baseline mTSS	1.00	0.99	1.02	0.319
Baseline CDAI	**1.03**	**1.00**	**1.05**	**0.038**
Baseline joint erosions (positive vs negative)	1.44	0.80	2.59	0.224

Odds ratios were estimated using a multivariate logistic regression model adjusted for baseline factors. Factors significantly associated with an increased or decreased risk of structural damage progression are shown in bold

*ACPA* anti-citrullinated protein antibody, *BMI* body mass index, *CDAI* Clinical Disease Activity Index, *CI* confidence interval, *HAQ-DI* Health Assessment Questionnaire-Disability Index, *hsCRP* high-sensitivity C-reactive protein, *mTSS* van der Heijde-modified total Sharp score, *MTX* methotrexate, *RA* rheumatoid arthritis, *RF* rheumatoid factor

## Discussion

The results of analyses from the phase 3 RA-BEGIN study showed that approximately 40% of patients with active RA and no or limited prior DMARD treatment achieved a sustained DAS28-hsCRP ≤ 3.2 or SDAI score ≤ 11 with baricitinib monotherapy 4 mg or baricitinib 4 mg plus MTX, as demonstrated in heatmaps of individual patient responses. Most of these patients achieved DAS28-hsCRP < 2.6 after 16–24 weeks. Patients were less likely to experience structural damage progression at week 52 if they achieved a sustained DAS28-hsCRP ≤ 3.2 or SDAI score ≤ 11 during the first 6 months of treatment (at weeks 16, 20, and 24), and in such patients, baricitinib 4 mg given as monotherapy or in combination with MTX was more effective than MTX monotherapy at reducing the risk of structural progression.

A cut-off value of < 3.2 for DAS28(CRP) was previously recommended by the ACR [18] and was also commonly used in clinical practice, clinical trials, and research publications [22–25] for differentiating LDA from moderate or more severe disease activity. However, it has become apparent over recent years that the values for remission and LDA as defined using DAS28 based on erythrocyte sedimentation rate (< 2.6 and < 3.2, respectively) are not accurate for DAS28(CRP). The cut-off value of ≤ 3.2 for DAS28-hsCRP in this study was selected when the protocol was initially designed, before it was understood that LDA according to DAS28(CRP) is actually lower than 3.2 [16, 17]. The cut-off value of ≤ 11 for SDAI was that recommended by the ACR [18, 19] and EULAR [20, 21] for defining LDA. It is important to note that, although achieving DAS28-hsCRP ≤ 3.2 or an SDAI score ≤ 11 was used as the cut off, we are not suggesting that this should be the treatment target, especially in patients who have previously received minimal treatment for RA. As recommended by EULAR and the ACR, the primary treatment target should be remission, with low disease activity being the target only if achieving remission is not feasible, using either Boolean remission or SDAI score ≤ 3.3 [19, 20]. The results of these *post hoc* analyses suggest that patients achieving DAS28-hsCRP ≤ 3.2 or a SDAI score ≤ 11 are less likely to have structural damage progression than patients who do not achieve these targets, suggesting that lower disease activity is important to preserve structural integrity.

Identification of baseline factors associated with an increased risk of structural damage progression in patients initiating therapy may help clinicians to identify those who might be at greatest risk. Baseline factors identified in the current study included higher hsCRP, a higher CDAI score, smoking, lower BMI, and female sex, suggesting that male patients and non-smokers are at lower risk of structural damage progression than female patients and smokers. This is in line with the fact that female sex and smoking are known risk factors for RA [25]. A number of other studies have investigated factors associated with increased structural damage in early RA. These included the presence of RF and/or anti-citrullinated protein antibodies, low haemoglobin levels, decreased bone mineral density, the presence of magnetic resonance imaging bone marrow oedema, the presence of tumour necrosis factor-α 308A allele with increased metalloproteinase 3 activity, female sex, and local joint swelling with or without tenderness at least once in the first 2 years of the disease [1, 26–31]. In addition, high BMI was associated with a lower risk of structural damage progression [27].

However, in contrast to our findings, results of one study suggested that male sex is associated with a higher risk of structural damage progression [27], whereas another report suggested there is no difference between the sexes in joint damage [32]. A recent report questioned the association with smoking, as the authors found no difference in joint damage between smokers and non-smokers [33].

Our findings are limited in that the analyses were post hoc*.* Nevertheless, we included data from a large number of patients and conducted multivariate analyses to control for potential imbalances in baseline factors. Two measures of sustained clinical improvement were used, and both analyses produced similar findings.

In conclusion, although no formal statistical comparisons were performed between treatments with respect to the proportion of patients with structural damage progression based on treatment response, our results indicate that patients with active RA who achieved a sustained DAS28-hsCRP ≤ 3.2 or SDAI score ≤ 11 with treatment are probably less likely to experience structural damage progression than patients who do not, independent of treatment. In patients with a sustained DAS28-hsCRP ≤ 3.2 or SDAI score ≤ 11, structural damage progression was more likely with MTX monotherapy than with baricitinib, either as monotherapy or in combination with MTX. In patients who did not achieve a sustained DAS28-hsCRP ≤ 3.2 or SDAI score ≤ 11, structural damage progression was less likely with a combination of baricitinib plus MTX than with MTX monotherapy. For these patients, the clinician should consider alternative treatment to enable the patient to achieve the recommended treatment goals of remission or low disease activity. Independent of treatment, baseline factors significantly associated with increased risk of structural damage progression included higher hsCRP and CDAI score, smoking, female sex, and lower body mass index.

## Electronic supplementary material

ESM 1 (PDF 915 kb)

ESM 2 (PDF 126 kb)

ESM 3 (PDF 1016 kb)
