# Supplementary material for: Structural damage progression in patients with early rheumatoid arthritis treated with methotrexate, baricitinib, or baricitinib plus methotrexate based on clinical response in the phase 3 RA-BEGIN study
Source: Clin Rheumatol. 2018 Aug 4;37(9):2381–90. doi: 10.1007/s10067-018-4221-0 (PMC6097080; doi:10.1007/s10067-018-4221-0)

Online Resource 1

Article title: Structural damage progression in patients with early rheumatoid arthritis treated with methotrexate, baricitinib or baricitinib plus methotrexate based on clinical response in the phase 3 RA-BEGIN study

Journal: *Clinical Rheumatology*

Authors: Désirée van der Heijde, Patrick Durez, Georg Schett, Esperanza Naredo, Mikkel Østergaard, Gabriella Meszaros, Francesco De Leonardis, Inmaculada de la Torre, Pedro López-Romero, Douglas Schlichting, Eric Nantz, Roy Fleischmann

Affiliation of corresponding author: Leiden University Medical Center, Leiden, The Netherlands, email address: mail@dvanderheijde.nl

Legend: Heatmaps showing individual DAS28-hsCRP responses to treatment in **(a)** DAS28-Group A (sustained DAS28-hsCRP ≤3.2 at weeks 16, 20 and 24) and **(b)** DAS28-Group B (DAS28-hsCRP >3.2 or missing data at any of weeks 16, 20 and 24).

Bari baricitinib, *base* baseline, *DAS28* Disease Activity Score for 28-joint counts, *hsCRP* high-sensitivity C-reactive protein, *MTX* methotrexate, *NA* not available, *w* week

DAS28-hsCRP colour codes: ≤2.6 | >2.6 to ≤3.2 | >3.2 to ≤5.1 | >5.1 | NA

(a) **MTX (n=45)** **Bari (n=67)** **Bari + MTX (n=100)**

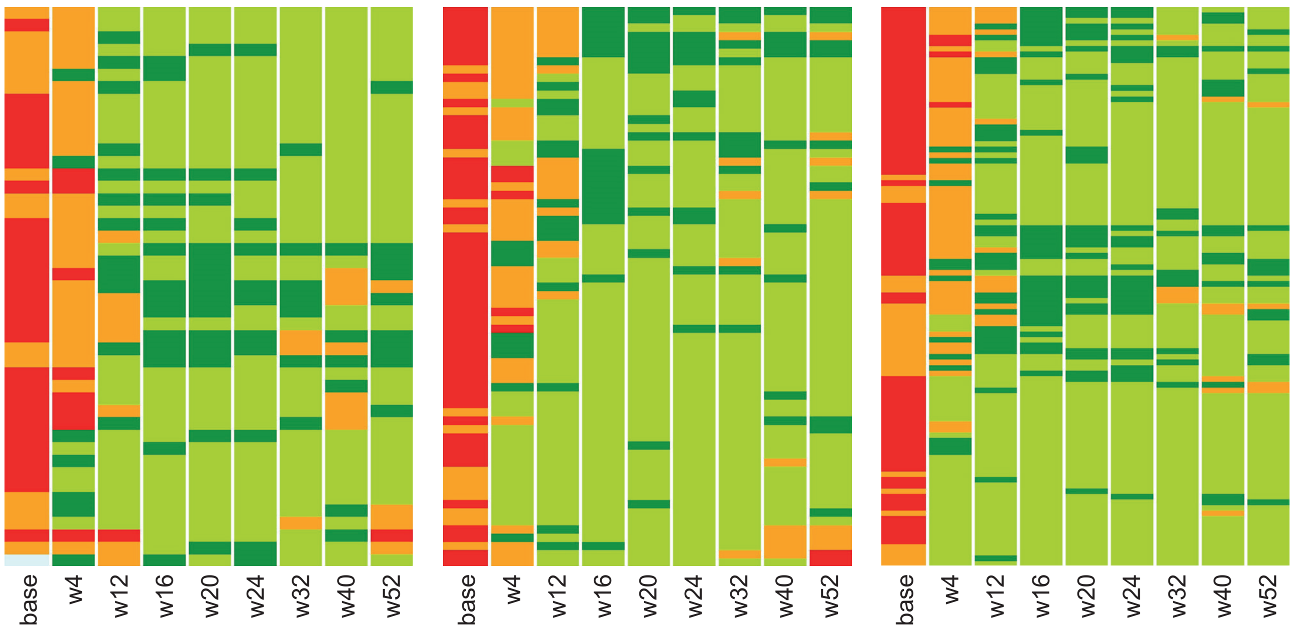

(b) **MTX (n=165)** **Bari (n=92)** **Bari + MTX (n=115)**

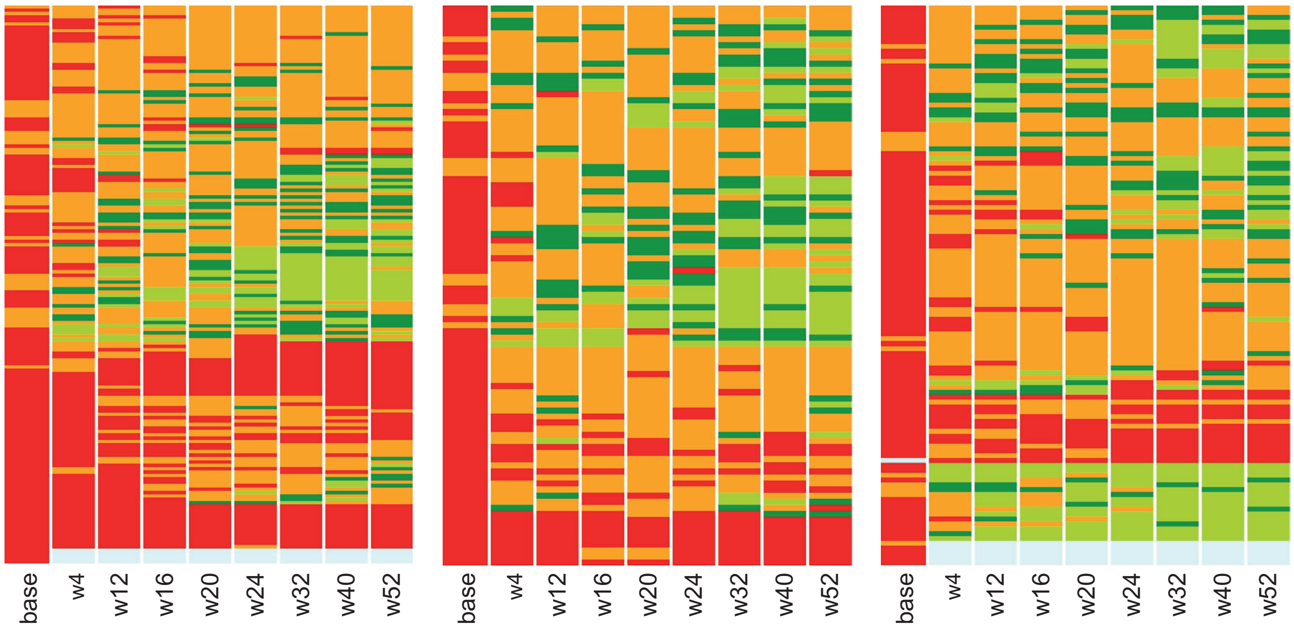

Supplement: Supplementary file 1 — (PDF 915 kb) [file 10067_2018_4221_MOESM1_ESM.pdf]
