# Supplementary material for: Structural damage progression in patients with early rheumatoid arthritis treated with methotrexate, baricitinib, or baricitinib plus methotrexate based on clinical response in the phase 3 RA-BEGIN study
Source: Clin Rheumatol. 2018 Aug 4;37(9):2381–90. doi: 10.1007/s10067-018-4221-0 (PMC6097080; doi:10.1007/s10067-018-4221-0)
Supplement: Supplementary file 2 — (PDF 126 kb) [file 10067_2018_4221_MOESM2_ESM.pdf]

Online Resource **2**

Article title: Structural damage progression in patients with early rheumatoid arthritis treated with methotrexate, baricitinib or baricitinib plus methotrexate based on clinical response in the phase 3 RA-BEGIN study

Journal: *Clinical Rheumatology*

Authors: Désirée van der Heijde, Patrick Durez, Georg Schett, Esperanza Naredo, Mikkel Østergaard, Gabriella Meszaros, Francesco De Leonardis, Inmaculada de la Torre, Pedro López-Romero, Douglas Schlichting, Eric Nantz, Roy Fleischmann

Affiliation of corresponding author: Leiden University Medical Center, Leiden, The Netherlands, email address: mail@dvanderheijde.nl

Patient baseline characteristics grouped by SDAI score and treatment (SDAI-Group A: sustained SDAI score ≤11 at weeks 16, 20 and 24; SDAI-Group B: SDAI score >11 or missing data at any of weeks 16, 20 and 24)

**Treatment**	**SDAI-Group A (N=209)**			**SDAI-Group B (N=375)**		
**(N; % of treatment group)**	**MTX (N=53; 25.2%)**	**Bari (N=62; 39.0%)**	**Bari + MTX (N=94; 43.7%)**	**MTX (N=157; 74.8%)**	**Bari (N=97; 61.0%)**	**Bari + MTX (N=121; 56.3%)**
Age, years	52 ± 13	51 ± 13	45 ± 14	50 ± 14	51 ± 13	51 ± 13
Female	36 (67.9)	47 (75.8)	67 (71.3)	112 (71.3)	74 (76.3)	89 (73.6)
BMI, kg/m^2	26.3 ± 6.0	27.7 ± 7.4	25.7 ± 5.1	26.6 ± 6.3	26.3 ± 6.1	27.3 ± 6.5
Smoker	10 (18.9)	17 (27.4)	20 (21.3)	38 (24.2)	20 (20.6)	27 (22.3)
Duration of RA from diagnosis, years[a]	0.2 (0.1, 0.4)	0.2 (0.1, 0.4)	0.2 (0.1, 1.0)	0.2 (0.1, 0.7)	0.4 (0.1, 1.6)	0.2 (0.1, 1.1)
ACPA positive[b]	48 (90.6)	59 (95.2)	88 (93.6)	145 (92.9)	83 (85.6)	104 (86.0)
RF positive[c]	50 (94.3)	61 (98.4)	88 (93.6)	153 (97.5)	94 (96.9)	116 (95.9)
≥1 joint erosion	35 (66.0)	41 (66.1)	52 (55.3)	103 (66.0)	64 (66.0)	85 (70.2)
hsCRP, mg/L	16.4 ± 12.7	19.4 ± 19.2	25.7 ± 28.4	24.4 ± 23.8	26.5 ± 29.6	23.2 ± 30.3
DAS28-hsCRP	5.7 ± 0.9	5.6 ± 0.9	5.8 ± 0.9	5.9 ± 1.0	6.1 ± 1.0	6.0 ± 0.9
SDAI	39.7 ± 12.9	38.2 ± 12.4	40.9 ± 12.8	42.3 ± 14.2	45.6 ± 14.2	44.2 ± 13.6
mTSS	9.7 ± 21.8	10.5 ± 25.5	7.5 ± 14.7	12.6 ± 22.4	15.2 ± 28.0	14.9 ± 23.5
HAQ-DI total score	1.5 ± 0.7	1.5 ± 0.8	1.6 ± 0.7	1.7 ± 0.7	1.7 ± 0.7	1.6 ± 0.6

Data are reported as mean ± standard deviation or n (%) unless otherwise indicated

[a]Data presented as medians with interquartile range

[b]ACPA positive (>ULN [ULN=10 U/mL])

[c]RF positive (>ULN [ULN=14 U/mL])

ACPA anti-cyclic citrullinated peptide antibody, *Bari* baricitinib, *BMI* body mass index, *DAS28* Disease Activity Score for 28-joint counts, *HAQ-DI* Health Assessment Questionnaire-Disability Index, *hsCRP* high-sensitivity C-reactive protein, *mTSS* van der Heijde-modified total Sharp score, *MTX* methotrexate, *RA* rheumatoid arthritis, *RF* rheumatoid factor, *SDAI* Simplified Disease Activity Index, *ULN* upper limit of normal
