# Supplementary material for: Structural damage progression in patients with early rheumatoid arthritis treated with methotrexate, baricitinib, or baricitinib plus methotrexate based on clinical response in the phase 3 RA-BEGIN study
Source: Clin Rheumatol. 2018 Aug 4;37(9):2381–90. doi: 10.1007/s10067-018-4221-0 (PMC6097080; doi:10.1007/s10067-018-4221-0)

Online Resource 3

Article title: Structural damage progression in patients with early rheumatoid arthritis treated with methotrexate, baricitinib or baricitinib plus methotrexate based on clinical response in the phase 3 RA-BEGIN study

Journal: *Clinical Rheumatology*

Authors: Désirée van der Heijde, Patrick Durez, Georg Schett, Esperanza Naredo, Mikkel Østergaard, Gabriella Meszaros, Francesco De Leonardis, Inmaculada de la Torre, Pedro López-Romero, Douglas Schlichting, Eric Nantz, Roy Fleischmann

Affiliation of corresponding author: Leiden University Medical Center, Leiden, The Netherlands, email address: mail@dvanderheijde.nl

Legend: Heatmaps showing individual SDAI responses to treatment in
(a) SDAI-Group A (sustained SDAI score ≤11 at weeks 16, 20 and 24) and
(b) SDAI-Group B (SDAI score >11 or missing data at any of weeks 16, 20 and 24).

Bari baricitinib, *base* baseline, *SDAI* Simplified Disease Activity Index, *MTX* methotrexate, *NA* not available, *w* week

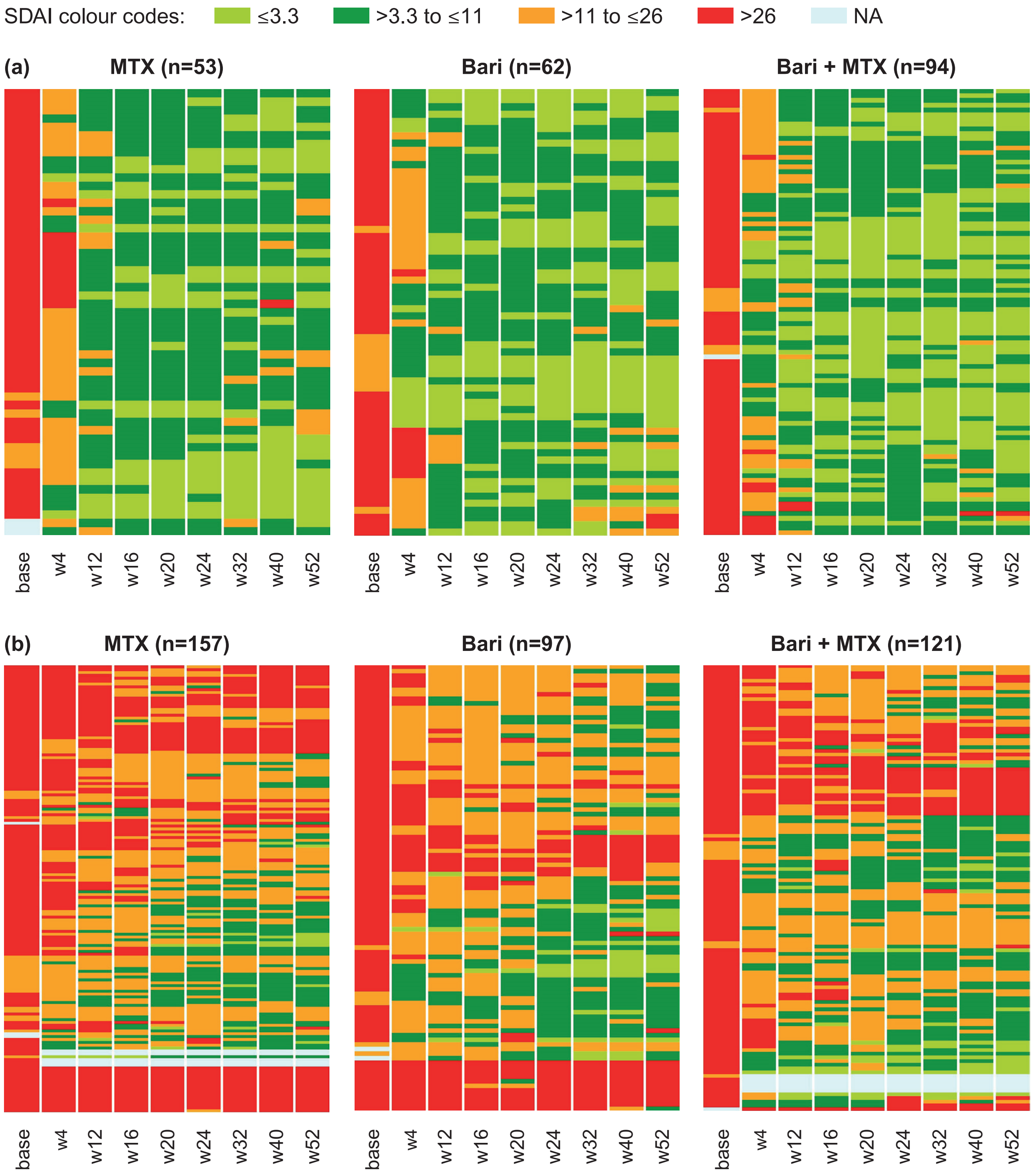

Supplement: Supplementary file 3 — (PDF 1016 kb) [file 10067_2018_4221_MOESM3_ESM.pdf]
